# The risk factor profile of women with secondary infertility: an unmatched case-control study in Kigali, Rwanda

**DOI:** 10.1186/1472-6874-11-32

**Published:** 2011-06-24

**Authors:** Nathalie Dhont, Stanley Luchters, Claude Muvunyi, Joseph Vyankandondera, Ludwig De Naeyer, Marleen Temmerman, Janneke van de Wijgert

**Affiliations:** 1 International Centre for Reproductive Health, Ghent University, Ghent, Belgium; 2 Projet Ubuzima, Kigali, Rwanda; 3 Ghent University, Microbiology Department, Belgium; 4 Microbiology Department, National University of Rwanda, Butare, Rwanda; 5 Belgian Technical Cooperation, Kigali, Rwanda; 6 Management Sciences for Health, Kigali, Rwanda; 7 Department of Internal Medicine, Academic Medical Centre of the University of Amsterdam, Amsterdam, the Netherlands; 8 Amsterdam Institute for Global Health and Development, Amsterdam, the Netherlands

## Abstract

**Background:**

Secondary infertility is a common, preventable but neglected reproductive health problem in resource-poor countries. This study examines the association of past sexually transmitted infections (STIs) including HIV, bacterial vaginosis (BV) and factors in the obstetric history with secondary infertility and their relative contributions to secondary infertility.

**Methods:**

Between November 2007 and May 2009 a research infertility clinic was set up at the Kigali University Teaching Hospital in Rwanda. Cases were defined as sexually-active women aged 21-45 years presenting with secondary infertility (n = 177), and controls as multiparous women in the same age groups who recently delivered (n = 219). Participants were interviewed about socio-demographic characteristics and obstetric history using structured questionnaires, and were tested for HIV and reproductive tract infections (RTIs).

**Results:**

Risk factors in the obstetric history for secondary infertility were lack of prenatal care in the last pregnancy, the first pregnancy before the age of 21 years, a history of unwanted pregnancy, a pregnancy with other than current partner, an adverse pregnancy outcome, stillbirth, postpartum infection and curettage. Presence of HIV, herpes simplex virus type 2 (HSV-2), or *Treponema pallidum *antibodies, and bacterial vaginosis (BV), were significantly more common in women in secondary infertile relationships than those in fertile relationships. The population attributable fractions (PAF%) for obstetric events, HIV, other (STIs), and BV were 25%, 30%, 27%, and 14% respectively.

**Conclusions:**

The main finding of this study is that obstetric events, HIV and other STIs contribute approximately equally to secondary infertility in Rwanda. Scaling up of HIV/STI prevention, increased access to family planning services, improvement of prenatal and obstetric care and reduction of stillbirth and infant mortality rates are all likely to decrease secondary infertility in sub-Saharan Africa.

## Background

In clinical medicine, secondary infertility is usually defined as the inability to conceive despite exposure to pregnancy for one year (2 years in some epidemiological studies), after having conceived at least once before [1]. This implies that women with secondary infertility do not necessarily have a living child. Studies looking at prevalence of infertility have all used slightly different definitions making comparison and overall estimates of secondary infertility difficult [2]. Nevertheless, it is well established that primary infertility is much more common than secondary infertility in resource-rich countries but that the reverse is true in sub-Saharan Africa (SSA) [1,3]. In certain African regions, the prevalence of secondary infertility is more than 30% [4]. The high secondary infertility rate in SSA is thought to be due to sexually transmitted infections (STIs) and medical interventions under unhygienic conditions, particularly during delivery or induced abortions [5]. Very few studies have actually examined risk factors for secondary infertility in SSA and little is known about the relative contribution of each factor. In a case-control study in Nigeria, induced abortion, postpartum infection, manual removal of the placenta and prolonged unsupervised labour were associated with secondary infertility [6]. In an analysis of Demographic Health Surveys (DHS) from different Central-African countries risky sexual behaviour such as multiple unions, transactional sex and early age at first intercourse were associated with secondary infertility [7].

Secondary infertility, although not life-threatening, can have severe consequences for the couples involved, especially for the women. If a woman cannot produce a living child in a formal union with a man, she will often be told to leave, and is isolated and stigmatized in the wider community [8-13]. Currently, very few of these women have access to effective infertility treatment. Reproductive technologies are either not available or prohibitively expensive in most SSA countries, although some medical professionals strive to make these techniques more affordable and accessible in SSA in the future [14]. Since most of secondary infertility is preventable, a better knowledge of modifiable risk factors may help to develop interventions targeted at those most at risk.

In order to shed more light on risk factors for secondary infertility in SSA and their relative contribution, we conducted a case control study in Kigali, Rwanda, comparing HIV and other reproductive tract infections (RTIs), past contraceptive behaviour and obstetric history among women in secondary infertile relationships and multiparous fertile women who recently delivered.

## Methods

Between November 2007 and May 2009, an infertility research clinic was opened at the Kigali University Teaching Hospital in Rwanda, which is the largest public hospital and the main referral centre for infertility pathology in Rwanda. Women in infertile relationships were recruited mainly through word of mouth. To be eligible for participation, women needed to be between 21 and 45 years of age, residing in Kigali, willing to undergo HIV testing and having had sexual intercourse at least once in the last 2 weeks. Refusal to participate in the study did not influence access to further services. Secondary infertility was defined as having had regular unprotected intercourse for one year or more with at least one regular partner without conception in women who conceived at least once before.

Fertile controls were defined as currently non-pregnant women who recently (between 6 to 18 months ago) delivered. Controls were recruited at the level of the community, as no appropriate control group could be selected at the hospital. A list of all residential neighbourhoods (known as umudugudu) from where the infertile couples originated was compiled (120 umudugudus in total). Fourteen neighbourhoods were randomly selected from this list by blindly hand-picking numbers from a bowl. Community mobilisers in these neighbourhoods visited all families to identify potentially eligible women. The eligibility criteria were the same as for women in infertile relationships, except for fertility status.

All participating women were offered free cervical cancer screening and treatment when clinically indicated.

The study was approved by the National Ethics Committee of Rwanda and the Ethics Committee of the Ghent University Hospital.

The purpose of the study was explained to potential participants in the local language (Kinyarwanda) and eligibility criteria were checked. All participants provided written informed consent. Socio-demographic characteristics and information on contraceptive practices and obstetric history were elicited in face to face interviews using a structured questionnaire. All female participants received a pelvic examination by the same trained nurse, which was supervised by a gynaecologist. During the speculum exam, vaginal swabs were collected for the preparation of a wet mount, potassium hydroxide testing and pH testing. Saline wet mounts were examined in the study clinic for the presence of clue cells. Thereafter, the study nurse collected blood samples. These were aliquoted, and either tested on site for HIV and syphilis, or frozen and stored at -80°C until further analysis. HIV testing was conducted according to the national algorithm (Determine Rapid HIV-1/2 Test kits (Abbott Laboratories, Abbott Park, IL), followed by Uni-Gold Rapid Test (Trinity Biotech Plc, Ireland) if Determine-positive, and the Capillus HIV-1/HIV-2 Rapid Test (Trinity Biotech Plc, Ireland) if a tiebreaker was needed. *Treponema pallidum*-specific rapid test (SD Bioline Syphilis 3.0) was conducted on site, followed by RPR titration (Syphilis RPR test, Human, Germany) in the National Reference Laboratory of Rwanda. A positive Treponema-specific rapid test with a negative RPR was interpreted as a past, cured infection. Participants were advised to return to the study clinic for test results and, if required, received treatment according to local guidelines. Stored specimens were analysed for HSV-2 antibodies (Herpeselect 2 ELISA IgG, Focus Diagnostics) in the National Reference Laboratory of Rwanda, and for *Chlamydia trachomatis*-specific IgG antibodies by a synthetic-peptide based EIA test (labsystems, Helsinki, Finland) at Ghent University Hospital, Department of Microbiology. All participants were offered HIV counselling and same-day HIV testing; those testing positive were enrolled in an HIV care and treatment clinic of their choice. Women in infertile relationships received a tubal patency test using hysterosalpingography (HSG) in most and laporoscopy in some cases. Their male partners were offered semen analysis.

All women regardless of study group who had reported an unwanted pregnancy in the past were invited for a semi-structured in-depth interview to collect data about the circumstances in which the unwanted pregnancy had occurred.

Data collected during interviews and laboratory investigations were single entered and rigorously verified and cleaned using MS Access 2000 (Microsoft, Seattle, USA). Intercooled Stata 9.2 (Stata Corporation, College Station, Texas, USA) was used for statistical analysis of the data.

The cause of the secondary infertility was determined to be a male factor in some relationships, but a female factor in most. Some female factors may have been missed because investigations to determine the cause of infertility were not complete in all participants. Furthermore, the sensitivity of HSG to detect tubal pathology is only 65% [15]. We therefore included all women in secondary infertile relationships in these analyses, regardless of whether a male or female cause was identified.

In analyses including obstetric events as risk factors, we did not consider events that occurred during the last pregnancy or delivery for the fertile women, because it is unclear whether the fertile women were in fact still fertile during the time period since their last pregnancy or delivery. For this same reason, fertile primiparae were excluded. Furthermore, fertile women with a history of trying for the previous pregnancy for more than one year were considered subfertile and were excluded from the analysis.

Socio-demographic characteristics of women in infertile and fertile relationships were compared using Chi-squared tests or the Mann-Whitney *U*-test. The relationship between selected covariates and secondary infertility were examined and controlled for age using logistic regression.

RTIs and obstetric events can lead directly to infertility. Their independent associations with secondary infertility were examined in a multivariable logistic regression model. Risk factors were grouped according to the type of infection they represent: endogenous infection (bacterial vaginosis (BV)), iatrogenic infection (a composite variable including a history of curettage post-abortion or postpartum, unsafe abortion, caesarean section, and/or postpartum infection) and STIs (a composite variable including herpes simplex type 2 (HSV-2), syphilis, and/or chlamydial infection). HIV was kept as a separate risk factor.

Population Attributable Fractions (PAF%) were calculated for selected risk factors using the following formula: pe (AOR-1)/AOR with pe the proportion of cases that is exposed to risk factor.

## Results

Of 339 women in infertile relationships who presented at the study clinic, 312 were confirmed eligible for the study and enrolled. Among these, 177 women had conceived at least once before and were considered to have secondary infertility. Of 407 women in fertile relationships identified in the community, 352 (86%) came to the clinic and 312 eligible women were enrolled. Educational level and occupation did not differ between responders and non-responders. Of the 312 fertile women enrolled in the control group, 29 reported to have tried more than one year for the previous pregnancy and 64 had only had one pregnancy in the past, leaving 219 fertile women for this analysis. In 114 (71%) of the 161 women in secondary infertile relationships who completed infertility investigations a tubo-peritoneal factor was diagnosed on HSG or laparoscopy.

Women in secondary infertile relationships reported a total of 303 pregnancies, and fertile women a total of 625 pregnancies. Women in secondary infertile relationships were more likely to be older and to have had a deceased child than women in fertile relationships (Table 1). Thirteen percent of women in secondary infertile relationships were nulliparous (never carried a pregnancy beyond 24 weeks), 70% had had not more than one pregnancy and 44% had no living children.

**Table 1:** socio-demographic characteristics and reproductive history of cases and controls

Variable	cases N = 177 n (%)	controls N = 219 n (%)	*P*-value
**Total number of pregnancies^1^**	303	625	

**Pregnancies**, median (IQR)	1 (1-2)	3 (2-5)	< 0.001

**Year of last delivery**, median (IQR)	1998 (1994-2002)	2003 (1999-2005)	< 0.001

**Age**, median (IQR)	32 (28-37)	28 (25-32)	< 0.001

**Education**			
Up to primary	135 (76)	175 (80)	0.38
More than primary	42 (24)	44 (20)	

**Marital status**			
Single	94 (53)	125 (57)	
Married	83 (47)	93 (42)	0.47
Separated, divorced or widowed	0 (0)	1 (1)	

**Parity**			
0	24 (13)	0 (0)	
1	101 (57)	9 (4)	< 0.001
2-3	45 (25)	118 (54)	
4 or more	7 (4)	92 (42)	

**Living children**			
0	78 (44)	0 (0)	< 0.001
1-2	93 (52)	84 (38)	
3 or more	6 (3)	135 (62)	

**Ever had a deceased child**			
No	94 (53)	154 (70)	< 0.001
yes	83 (47)	65 (30)	

^1^excluding last pregnancy for fertile women

Women in secondary infertile relationships were more likely to have had the first pregnancy before the age of 21 years (aOR = 2.56, CI = 1.63-4.02), an unwanted pregnancy (aOR = 11.51, CI = 5.47-24.20), a pregnancy with someone other than the current partner (aOR = 5.68, CI = 3.56-9.08), no prenatal care in the last pregnancy (aOR = 4.68, CI = 1.81-12.12), an adverse pregnancy outcome (aOR = 1.89, CI = 1.17-3.04), a stillbirth (aOR = 7.52, CI = 2.97-19.01) a postpartum infection (aOR = 11.49, CI = 3.31-39.89) and a curettage (aOR = 1.71, CI = 0.93-3.13) than women in fertile relationships (Table 2). Very few women reported to ever have had an intrauterine device fitted (five controls and two cases). The only studied obstetrical antecedents that were not associated with secondary infertility were an unattended birth and a caesarean section in the past.

**Table 2:** Association of obstetrical and reproductive history and reproductive tract infections with secondary infertility

Variable	Cases N = 177 n (%)	Controls N = 219 n (%)	Age adjusted OR
**Obstetrical and reproductive history**			
Ever IUCD	2 (1)	5 (2)	0.38 (0.07-2.10)
First pregnancy before age 21 years	97 (55)	92 (42)	2.56 (1.63-4.02)
Unwanted pregnancy	49 (28)	11 (5)	11.51 (5.47-24.20)
Pregnancy with another partner	105 (59)	48 (22)	5.68 (3.56-9.08)
No prenatal care in last pregnancy^1^	20 (15)	7 (4)	4.68 (1.81-12.12)
Unattended birth	44 (25)	75 (34)	0.81 (0.56-1.18)
Adverse pregnancy outcome^2^	57 (32)	44 (20)	1.89 (1.17-3.04)
Stillbirth	32 (18)	6 (3)	7.52 (2.97-19.01)
Unsafe abortion	3 (2)	0 (0)	1.33 (0.73-2.39)
Caesarean section	30 (17)	27 (12)	11.49 (3.31-39.89)
Postpartum infection	24 (13)	3 (1)	1.71 (0.93-3.13)
Curettage^3^	32 (18)	23 (10)	

**Reproductive tract infections**			
BV	50 (28)	32 (15)	2.68 (1.58-4.54)
Positive HIV serology	74 (42)	35 (16)	4.10 (2.50-6.72)
Positive HSV-2 serology	121 (70)	99 (45)	2.56 (1.65-3.96)
Positive Chlamydia serology	31 (18)	33 (15)	1.58 (0.89-2.80)
Old treated syphilis	16 (9)	8 (4)	2.85 (1.13-7.18)

^1 ^women whose last pregnancy was a miscarriage are excluded from analysis, leaving 129 cases and 195 controls

^2^includes miscarriage and ectopic pregnancy

^3^includes post-abortion and postpartum curettage

The presence of HIV, HSV-2, or *Treponema pallidum *antibodies was significantly more common in women in secondary infertile relationships than women in fertile relationships (aOR = 4.10, 95%CI = 2.50-6.72; aOR = 2.56, 95%CI = 1.65-3.96; aOR = 2.85, 95%CI = 1.13-7.18, respectively). The presence of *Chlamydia trachomatis *antibodies was higher in cases (31/177, 18%) than controls (33/219, 15%) but did not reach statistical significance (p = 0.12). Women in secondary infertile relationships were more likely to have a diagnosis of BV than women in fertile relationships (aOR = 2.68, 95%CI = 1.58-4.54). The PAF% for obstetric events, HIV, other sexually transmitted infections (STIs), and BV were 25%, 30%, 27%, and 14% respectively (Table 3).

**Table 3:** Population attributable fractions of direct risk factors for secondary infertility

predictor	Pe	AOR^3^	PAF%^4^
Obstetric events^1^	0.38	3.03	25
STIs ^2^	0.74	1.57	27
Endogenous infections (BV)	0.28	1.95	14
HIV	0.42	3.58	30

^1^includes postpartum infection, caesarean section, unsafe abortion and/or curettage

^2^includes positive HSV-2, Chlamydia and/or syphilis serology

^3 ^logistic regression model included all listed (composite) variables

^4 ^PAF is calculated using the following formula: PAF = pe(AOR-1)/AOR, with pe the proportion of cases exposed and AOR is derived from a logistic regression model containing all the listed biological factors and age.

Of the 49 women in infertile relationships and the 11 fertile women who had reported an unwanted pregnancy, 19 and seven women respectively returned for the in-depth interviews. Two of the fertile women had tried for their previous pregnancy for more than one year and are therefore considered subfertile, resulting in 21 women who reported unwanted pregnancies in the infertile and five in the fertile group. Almost all (95%) unwanted pregnancies in women in infertile relationships, and 20% in fertile women were with another partner than the current partner. Age at conception was less than 20 years for half of the women in secondary infertile relationships and for one woman in fertile relationships. In eight women in infertile relationships and in none of the women in a fertile relationship, the unwanted pregnancy was a consequence of rape. Another frequently reported scenario in the group of women in infertile relationships (five cases) was a younger girl having unforced sex with an older man without being aware of the possibility of pregnancy. The unwanted pregnancies in the fertile women were all, apart from one, due to failure to access effective contraception with the current partner. Six women in infertile relationships and no woman in fertile relationships reported a postpartum infection after delivery. Of the women in infertile relationships with a past of unwanted pregnancies, 14 out of 18 (78%) had tubal abnormalities on the HSG.

## Discussion

The main finding of this study is that obstetric events, HIV and other STIs contribute approximately equally to secondary infertility in Rwanda. Furthermore, the study indentified some previously unknown obstetric history risk factors for secondary infertility such as a history of no prenatal care during the last pregnancy, early age of first pregnancy, unwanted pregnancies and stillbirths.

Infections acquired during a previous delivery were strongly associated with secondary infertility as reported by other authors. A history of dilatation and curettage, which was weakly associated with secondary infertility in our population, has been associated with pelvic inflammatory disease (PID) but not with infertility in other studies [16]. In our study, we did not find an association between caesarean section and secondary infertility, but this could be due to insufficient statistical power [6,17,18].

A history of stillbirth was strongly associated with secondary infertility in our study. Stillbirths can contribute to infertility through several mechanisms: First, they can be a marker for perinatal or ascending infections during strenuous labour; a history of stillbirth has been associated with PID in previous research [16]. Second, stillbirths and high infant mortality rates create a child deficit which is associated with self-reported fertility impairment [19]. The latter might also explain the association between no prenatal care in the previous pregnancy and secondary infertility in our population. In Rwanda, prenatal care usually involves screening and treatment of syphilis and malaria prophylaxis. These infections are important causes of stillbirths in developing countries [20]. The association between HIV infection and secondary infertility could also be explained by an increased occurrence of stillbirths and infant mortality among infected mothers but the relationship is much more complex.

Associations of infertility with HIV and other RTIs such as HSV-2, Chlamydia and BV in this population are discussed in detail elsewhere [21,22]. In summary, the data from these analyses indicate that HIV and HSV-2 could play a role in tubal pathogenesis. In the opposite direction, infertility can also lead to the acquisition of HIV and other STIs through increased extramarital sex. The fact that secondary infertile women are more likely to be HIV infected and to report lifetime high-risk sexual behaviour than primary infertile and fertile women suggests that this group of women may enter a vicious circle of unsafe sex, beginning from the start of sexual activity, leading to a first pregnancy (in many cases unplanned and often the last pregnancy), STIs and HIV infection, all of which can impair their fertility. Thus, the different aspects of reproductive ill health go hand in hand indicating a need for a more holistic approach towards reproductive health services in SSA.

An interesting finding in our study, and not yet reported, is the strong association of unwanted pregnancies with infertility irrespective of induced abortions. Very few infertile women in our study reported induced abortions despite the large number of reported unwanted pregnancies. In a study in Nigeria, induced abortion and post abortion sepsis were the most important risk factors for secondary infertility [6]. Induced abortions may have been underreported in our study. However, we conducted semi-structured in-depth interviews with 27 out of 63 women reporting an unwanted pregnancy, and only one woman admitted to an induced abortion that she had not reported in her first structured interview. On the one hand unwanted pregnancies could lead to infertility through lack of prenatal and obstetric care. On the other hand, the unsafe sex which has caused the unwanted pregnancy could also have caused the acquisition of HIV/STIs with secondary infertility as a consequence. Previous studies have demonstrated that women experiencing unwanted births are seeking prenatal care less frequently and are more likely to have a pregnancy complication (preterm birth, perinatal deaths and postpartum infections) [23]. We learned from our in-depth interviews that unwanted pregnancies in women in infertile relationships are often a result of unsafe sex (including sexual violence and sex of younger girl with an older man), and are more likely to result in postpartum infections, although the numbers are too small to draw firm conclusions. Regardless of the underlying mechanisms, more use of family planning methods and condoms is likely to decrease not only unwanted pregnancies but also infertility rates.

The results of our study may not be generalised to the general population of Kigali. We do not know the response rate among women in infertile relationships because they were recruited through word of mouth. However, the response rate of fertile women in the community was high, the educational level of responders and non-responders in each study group was similar, and baseline characteristics (such as educational level and marital status) did not differ between cases and controls.

The unit of analysis was individual women, and not pregnancies or deliveries. Since women in fertile relationships had experienced twice as many pregnancies/deliveries than women in infertile relationships, the contributions of pregnancy-related and obstetric events to secondary infertility were likely underestimated.

Finally, due to the case-control design of this study, temporal relationships between past STIs and HIV and infertility cannot be ascertained.

## Conclusion

Improved obstetric and prenatal care in addition to a better prevention and management of HIV/STIs will contribute considerably to the prevention of infertility in SSA. Furthermore, decreasing stillbirth and infant mortality rates and improving access to family planning, especially for young women, have also a role to play. Finally, educational campaigns should focus on the fact that sex without a condom can lead not only to infections and unwanted pregnancies but also to infertility [24].

## Competing interests

The authors declare that they have no competing interests.

## Authors' contributions

ND conceived, designed and executed the study, performed the statistical analysis and drafted the manuscript. SL assisted with data analysis and interpretation of data. CM carried out some of the lab tests. JV assisted with study design and execution of the study. LDN assisted with data analysis. MT assisted with study design and drafting of the manuscript. JvdW assisted with study design, data analysis, interpretation of data and drafting of the manuscript. All authors read and approved the final manuscript.

## Pre-publication history

The pre-publication history for this paper can be accessed here:

http://www.biomedcentral.com/1472-6874/11/32/prepub

## Aknowledgments

This study was funded by a PhD grant from the Flemish Interuniversity Council (VLIR-UOS) and by the European and Developing Countries Clinical Trials Partnership (EDCTP) through a project entitled: "Preparing for Phase III vaginal microbicide trials in Rwanda and Kenya: Preparedness studies, capacity building, and strengthening of medical referral systems". EDCTP cannot accept any responsibility for information or views expressed herein. The project also received funding from the "Walking Egg npo", Genk 2000.

We thank Rosette Busasa, Ammiel Gasarabwe and the Community Mobilizers for their dedication during data collection, Dr. Gilles Ndabisaya for programming of the database, and other members of the Projet Ubuzima team and the EDCTP microbicides project team for their contributions and support.
